# Conceptual framework for management or transmission of knowledge in companies: A systematic review

**DOI:** 10.3389/fpsyg.2023.1124650

**Published:** 2023-04-05

**Authors:** Cláudia Pereira, Catherine Delgoulet, Marta Santos

**Affiliations:** ^1^Center for Psychology of University of Porto, Faculty of Psychology and Education Sciences of University of Porto, Porto, Portugal; ^2^Center for Research on Work and Development (CRDT), Conservatoire National des Arts et Métiers, Paris, France

**Keywords:** knowledge transmission, knowledge management, human resources management, conceptual framework, systematic review

## Abstract

The literature on knowledge management as a broad area, typically studied under the scope of business management, and on knowledge transmission as a process, often studied under the scope of work psychology and ergonomics, although extensive, lacks a synthesis regarding the dimensions involved in knowledge management or transmission practices in workplaces. Thus, this study aims to systematize the existing programs for management or transmission of knowledge in workplaces and to develop a conceptual framework to support their design and implementation in this context. Thereto, the study resorts to the main scientific approaches that address knowledge management and knowledge transmission (business management and work psychology/ergonomics). The methodology followed a systematic review to identify and analyse the programs concerning the management or transmission of knowledge implemented in professional contexts, nearly from the beginning of the 21st century, considering the two scientific approaches. The analysis of the 28 articles shows what defines the implemented practices, their impact, and the role assumed by HR. The results demonstrate differences and similarities between the two approaches which guided the dimensions included in the conceptual framework. This study makes advances for both the scientific field, bringing different scientific discourses closer together by acknowledging their strengths; and for practitioners, through the possibility of improving the understanding of the application scope of the concepts of knowledge management and knowledge transmission, as well as supporting their action in workplaces.

## 1. Introduction

### 1.1. Managing knowledge in professional contexts

The international entities focused on the employment, work and health dimensions of the working population, recognize knowledge in workplaces as a strategy to tackle some of the global issues that challenge companies, promoting their development and growth (e.g., ILO, 2020). These entities draw attention to the challenges that companies face not only today, but also in a near future: age diversity management; shortage of resources in the labor market; longevity of workers in professional contexts; loss of a significant number of workers due to their retirement and possible loss of critical knowledge acquired with experience.

Considering this problematic, literature presents different perspectives and scientific fields that address workers' knowledge in professional contexts. Two main approaches emerge in the field of studies and practices concerning the issue of knowledge management in professional contexts.

The first one concerns the scientific field of Business Management, where literature shows that knowledge is intended to be extracted from the workers to promote organizational storage, therefore tending to be technological, as well as to be capitalized on individual performance (Argote and Ingram, 2000; Levallet and Chan, 2019).

The second field concerns the scientific field of Work Psychology and Ergonomics (under the scope of the theory of activity research and social sciences), where knowledge is acquired, shared and preserved through a closer look at the real work activity in professional contexts (Lacomblez et al., 2007). This perspective considers the workers' experience, as well as the conditions and constraints associated with carrying out the activity itself (Ledoux et al., 2012; Santos et al., 2019), and the multiple dimensions of learning and knowledge transmission instancies (Delgoulet et al., 2012; Thébault et al., 2012). However, in the searches carried out, we didn't find studies that cross these perspectives, making it difficult to perceive their similarities and/or differences. This poses a gap in the literature that can be address in regards to what can support the action of those who intend to develop practices in these two fields (academics or practitioners).

It is in the light of this scientific problem and lack of cross-fertilization of scientific views and professional practices on a shared theme that this article focuses its objective. It is an opportunity to clarify, in relation to these two approaches, the different ways in which knowledge is perceived and managed, the different involved practitioners and the principles associated with each practice. Thus, we put into perspective the relation between knowledge management, as a broad area studied mainly in the field of business management, and knowledge transmission, as a process which is most frequently studied in the field of work psychology and ergonomics.

Following this, our main research question is: what are the characteristics of the programs regarding the management or transmission of knowledge that have been implemented in professional contexts through the last 18 years? To explore this question, a systematic review was conducted. Based on the obtained results we propose a conceptual framework that organizes the dimensions involved in the design and implementation of programs of management or transmission of knowledge in workplaces. We reinforce that these are situated in the field of business management (knowledge management) and the field of work psychology/ergonomics (knowledge transmission). Although this work is not a historical review, but rather a contemporary approach to the topic, the framework crosses theoretical perspectives and focuses on its usefulness in workplaces in terms of supporting the action of practitioners.

The findings and discussion around this topic can support the reflection, among academics and practitioners, about what dimensions should be considered int the design and implementation of programs. Bringing scientific discourses closer together has the advantage of enhancing both the discussion and action in light of the global challenges that may arise.

### 1.2. Knowledge-related practices in professional contexts: Point of view of two scientific areas

Knowledge management and knowledge transmission are understood from the perspective of the scientific areas that often focus on the study of these concepts, and are based on distinct principles concerning the understanding of and dealing with workers' knowledge.

Thus, in the field of business management, it is known that knowledge management is associated with the ability to identify ways of creating, capturing and preserving knowledge, as well as recognizing its value and specificity, which are difficult to replace in a certain context, and capitalizing it on performance (Argote and Ingram, 2000; Calo, 2008). Often, knowledge management aims for the development of digital tools (e.g., intranets) that organize knowledge, creating a memory of the company (Wiig, 1997; Heisig, 2009) and enhancing individual and organizational performance (Levallet and Chan, 2019). It is understood, therefore, that knowledge management is an approach, a broad area, that can integrate different ways and strategies to create, capture or preserve knowledge (e.g., Zack, 1999; Choi et al., 2008; Venkitachalam and Willmott, 2017).

Due to the proximity between concepts and taking into account the objective of the study, it is important to mention the existence of three complementary concepts (referred in the literature and used in professional contexts), scientifically addressed as branches of the broad area of knowledge management and within the field of business management: (i) knowledge retention (KR), which focuses on the identification of knowledge, particularly critical knowledge, at risk of being lost with retiring employees, and on the implementation of strategies to preserve this knowledge in the organizations (Liebowitz, 2009); (ii) knowledge transfer (KT), that typically refers to an unidirectional logic that favors only one sender and one receiver, and where the transfer of knowledge does not occur naturally or spontaneously (Calo, 2008); (iii) knowledge sharing (KS), which is understood as a critical stage in knowledge transfer (using a personalisation strategy) and occurs at an individual level (unidirectional or bidirectional sharing) (Tangaraja et al., 2016). In this sense, it is also important to note that the literature in this scientific field assumes that knowledge transmission can also be a process within knowledge management practices (e.g., Schulz and Jobe, 2001). However, from the point of view of work psychology, this knowledge transmission is autonomized as an object of study and as a process mobilized in everyday life, and it is with this point of view that we present it next, as well as considering the scope of this study.

In the field of work psychology and ergonomics, knowledge transmission can be understood as a cognitive, social and organizational process, situated at work and proteiform (e.g., Delgoulet et al., 2012; Thébault et al., 2014), which can also integrate different strategies and ways in which knowledge can be transmitted and developed. This corresponds to an interaction between two or more people, in a given situation (Thébault, 2018) and implies the manifestation of the experience and its transformation into a dynamic process of action and co-constructed learning—for the newcomers and the experienced workers (Gaudart and Thébault, 2012). It is then perceived that the transmission can occur informally (on a daily basis, without support from HR teams) or *via* knowledge management mechanisms (Cloutier et al., 2012). Knowledge transmission expresses the relevant role workers play in the acquisition, sharing and reflection of knowledge in the workplace, especially tacit, critical knowledge, as well as prudence knowledge^1^, all acquired from experience and difficult to transmit (Diallo and Clot, 2003). In this context, various responses have been established, such as intergenerational learning programs, mentoring programs, systems to involve experts, or training activities that are articulated with the analysis of the work activity (e.g., Ropes, 2011; Santos et al., 2019).

Taking into consideration that the studies which support this analysis do not use the same exact terms and in order to facilitate the reference to knowledge management issues from the business management point of view, as well as to knowledge transmission from the work psychology and ergonomics point of view, we choose to refer to these concepts using the expression “management or transmission of knowledge”. Assuming that this expression considers both scientific perspectives favored in this study.

### 1.3. Role of Human Resources (HR)

Whatever the company's knowledge-related purpose may be, the literature shows that HR teams have an important role in these processes due to the nature of their function and given that they are essential for the development and sustainability of companies (Calo, 2008). These teams can contribute to the preservation of knowledge, within the context, and to a feeling of recognition by the workers involved in these processes (Calo, 2008). However, HR practices are found to be generally informal (Hamey and Alkhalaf, 2020) and these professionals may lack information to properly implement practices, in particular, on how to value workers' knowledge (Iles et al., 2001). Moreover, it is mentioned in both the scientific areas privileged in this study, that initiatives focused on dealing with knowledge of workers sometimes seem to be implemented without considering working conditions or the workers' perspectives; and, due to the demands and intensification of HR's own activity (spending time dealing with workers' issues; helping their team with tasks; see, e.g., Metz et al., 2014), these initiatives tend not to be followed up or maintained over time (Oltra, 2005).

## 2. Method

### 2.1. Research strategy

The research strategy and definition of the research questions were guided by the PICOS approach (Liberati et al., 2009), applied to the systematic review that was conducted and illustrated in Table 1 below.

**Table 1:** PICOS approach applied to the systematic review.

**Population**	**Workers from different professional contexts**
Interventions	Programs for management or transmission of knowledge carried out in professional contexts
Comparisons	(not applied in this research)
Outcomes	Characteristics of the programs for management or transmission of knowledge
Study design	Systematic review, through a search of empirical studies published over the last 18 years, with a qualitative approach

The systematic review considered the search of empirical studies, with a qualitative approach, carried out in the last 18 years (study design), integrating programs for management and transmission of knowledge carried out in professional contexts with workers (interventions; population). The outcomes to be obtained are the characteristics of these programs (e.g., what kind of practices and how they are implemented; impacts), in order to support the proposal of the conceptual framework for knowledge management or transmission. In the context of this study, we consider that the “comparisons” field from PICOS is not applicable in this type of research, since the objective of the study values the explanation of practices and aims to highlight relationships between them (not to analyse comparisons).

Considering the general research question presented in Section 1, the following specific research questions were defined, in order to obtain data that could be useful for the discussion on the subject and the conceptual framework to be proposed:

1. What are the global issues that sustain the pursuit of a knowledge management (including the branches of KR, KT, KS) or transmission program?
2. What are the main practices used for knowledge management or transmission?
3. Who conducts the programs and what role do those players assume?
4. What kind of knowledge is transmitted and how is it transmitted?
5. What is the duration of the practices' implementation?
6. What is the impact (benefits and limitations) of the implemented programs?

These specific questions guided the analysis of the articles integrated in the systematic review.

The article search was carried out in 2022, in accordance with the PRISMA methodology (Liberati et al., 2009) in two databases: SCOPUS and Emerald Insight databases. The choice for these databases relies on three arguments: (i) their scientific quality; (ii) the type of articles published (scientific and empirical articles), and; (iii) the thematic field covered in relation to management or transmission of knowledge.

The keywords are related to the definition of the scope of investigation, determined in the previous section. Four keywords are used to cover this perimeter: “knowledge retention program” OR “knowledge transmission program” OR “knowledge transfer program” OR “knowledge management program” OR “knowledge sharing program.”

The option to consider the word “program” was related to the need to only find articles that could illustrate a set of activities or practices followed for a particular purpose (in this case, the management or transmission of knowledge in a particular sector, team or company), and not to collect and analyse the vast scientific content about knowledge management or knowledge transmission.

Prior to defining the keywords, a dummy and initial search (Daniels, 2019) was conducted to verify whether there would be any systematic reviews already published on these issues. Moreover, it guides the definition of appropriate keywords to obtain the intended content, which is also relevant to the addressed scientific areas (Business Management; Work Psychology/Ergonomics). This dummy and initial search showed that it would not be possible to choose search keywords that would allow us to encompass studies with distant theoretical backgrounds. That is, studies related to the areas of Business Management, where the topic of management or transmission of knowledge is typically worked on, and studies related to the area of Work Psychology/Ergonomics, where this topic is also studied. This is due to the fact that the latter is part of a tradition of Activity Theory Research, where the type of analysis made on initiatives for management or transmission of knowledge does not necessarily contemplate the use of major concepts as keywords, although they are present in the studies. Thus, to ensure that the analysis and discussion of the topic could be complemented with this point of view, a search was also conducted through other sources: hand screening in journals that publish in the areas of Work Psychology and Ergonomics (e.g., Work; Management and Avenir) and other websites (e.g., research centers related to work issues). The analysis and inclusion of these other records were fundamental to allow for the consideration of other sources (from different scientific backgrounds) and to make the analyses and the review more integrative.

### 2.2. Inclusion and exclusion criteria

The article search was carried out considering the objective defined for the systematic review: to identify and analyse the programs regarding the management or transmission of knowledge carried out in professional contexts, over the last 18 years, to sustain the identification of the management or transmission of knowledge dimensions for the conceptual framework. The aim is to enhance the reflection between academics and to support the action of practitioners in companies, in particular Human Resources teams. The option for the analysis of the last 18 years is sustained by studies like Heisig (2009) that show two meaningful insights: (i) companies have started to demonstrate more explicit and organized concerns with this topic and with investing in their contexts, as of 2004/2005 approximately; and (ii) it was in 2004 that a European guide for good practices in knowledge management was published (CEN/European Committee for Standardization, 2004), thus being understood as a reference in the time period to consider in the systematic research. Therefore, these were the references taken into consideration to assume this period (2004) as the time frame from which articles could be found to match the needs of this research.

To operationalise the search, the inclusion criteria defined for the article search were: (i) articles published in peer-reviewed journals between 2004 and 2022, written in English or French; (ii) empirical articles, with a qualitative approach (by privileging the process, the description of activities and practices) that presents management or transmission programs (or similar, such as management or transmission initiatives, actions, practices); (iii) articles from the databases whose title or abstract included the search keywords; (iv) articles whose participants were workers in a professional context.

For the search of the keywords, the specificities of the search fields of both databases were also considered. That is, in the case of Emerald Insight, the available filters in the database for selection were: the type of material to search (journal articles); year of publication (2004–2022); and “access type” (all content). In the case of SCOPUS, since the platform allows the selection of other types of filters, the following were selected in order to refine the search: year of publication (2004–2022); disciplines (social sciences, business, psychology); type of document (journal/articles); keywords (the selection included all the available keywords that relate in some way to the keywords of the search, such as “knowledge management,” “knowledge transfer,” “knowledge,” “program development,” “qualitative analysis”); language of article (English, French); articles in open access. The search with the keywords was conducted first for “title” and second for “abstract,” due to the platforms' inability to enable selecting both simultaneously.

Articles from the databases that did not include, in their title or abstract, the keywords defined for the search, and whose methodology or results were not related to the intended theme, nor contributed towards meeting the objective of this systematic review (e.g., systematic review studies; studies conducted in an academic context) were excluded.

### 2.3. Number of selected articles and categories defined for analysis

**Figure 1:**
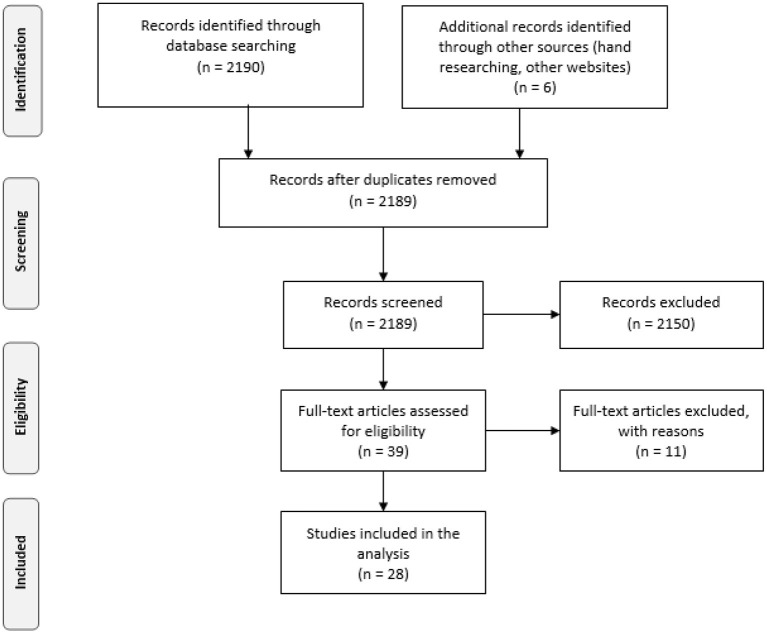
PRISMA flow diagram.

Based on the PRISMA methodology (Figure 1; Liberati et al., 2009), the search allowed us to identify 2,196 articles through database searching and other sources. The articles were found in the databases defined for the search (*n* = 2,190), as well as through hand screening and other websites (like *Work; Management and Avenir*; and other websites like research centers related to work issues) (*n* = 6). After the elimination of duplicates and a first reading of the abstracts, method and results of the articles, 2,189 articles were eliminated (for not meeting the inclusion criteria), leading to a total of 39 articles analyzed for eligibility. After this analysis, 11 studies were excluded: although in the screened records, they appeared to meet the inclusion criteria, it was clear from the full reading of the articles that their content made it unfeasible to analyse the articles for the intended purpose (e.g., articles where the qualitative approach provided descriptions and explanations that were not sufficient to understand the implemented knowledge management program). Thus, a total of 28 studies were included in the systematic review. The selected articles are summarized in Supplementary Table S1 and listed in the references with an asterisk (^*^).

For each of the selected studies, data were analyzed considering the research questions and according to the following categories: professional context, country of the study and scientific field that supports the development of the practices; types of issues that support the need to implement a program for knowledge management or transmission problem; used techniques and implemented initiatives for retention and transmission of knowledge and their duration; key interlocutors who implemented the initiatives; participants in the study; type of knowledge evidenced for management or transmission and how it was transmitted/managed; main impact of the initiatives. The results on these components support the elaboration of the conceptual framework proposed in the Discussion.

## 3. Results

### 3.1. Characterization of the implemented management or transmission programs

All 28 studies are of a qualitative nature and have been carried out in 13 countries, spread over almost all continents. They are distributed along different economic sectors of activity (mostly in Health, Industry and Other Services), corresponding to a total of 17 professional sectors. Table 2 shows the details of this distribution, providing a context of the analyzed articles. As a reference, the categorisation of sectors of economic activity used in the sixth European Working Conditions Survey was utilized (Eurofound, 2017).

**Table 2:** Characterization of articles by sector and country of the study (organized by sector of economic activity).

**Sector of economic activity**	**Country**	**Number of articles included**
Industry (manufacturing, poultry processing plant, electricity supplier, industry not classified)	Australia, Canada, France, Portugal, United States of America	Six
Transport (aviation)	Spain	One
Financial Services (financial and insurance)	Australia, Nigeria	Three
Public administration (law, police forces)	United Kingdom	Two
Health	Canada, France, Malaysia, Spain, United States of America	Five
Other services (information, telecommunications, arts, consulting, engineering, technological development)	France, Hong Kong, Indonesia, Malaysia, Spain, Switzerland, United Kingdom, United States of America	Nine
Diverse (cinematography; health; food services)	Canada	One
Unclassified	Germany	One

### 3.2. Global issues addressed by knowledge management or transmission and main practices applied

The main global issues that unroll the development of a knowledge management or transmission program (research question 1) are threefold: workforce demographic changes; maintaining a competitive advantage in professional markets; and the acceleration of workplace transformations.

Six types of issues that reveal the need to implement a program for knowledge management or transmission (in the contexts of the analyzed studies) were identified: (1) Keeping competitive advantage in fast changing markets, innovation and performance of company; (2) Changing demographics in the workplace; (3) Acceleration of workplace transformations and professional working techniques or models that are difficult to keep up daily by workers; (4) Concerns with reliability in tasks; (6) Concerns with health and safety of workers.

It should be noted that some of the programs were implemented for more than one reason (e.g., one given context has, simultaneously, demographic changes and technological working techniques transformations). This finding reveals the variability associated with the motives for investing in knowledge management or transmission.

Nine types of practices have been identified and are aligned with the scientific evidence on this issue (e.g., Ropes, 2011). Since articles from different disciplines were identified in the systematic review, the practices are presented according to their scientific background, in order to demonstrate proximities and specificities found between both areas in the use of different practices (Table 3). To refer to any of the articles analyzed in the systematic review, the numbering given in the Appendix is presented between brackets.

**Table 3:** Types of practices for management or transmission of knowledge identified in the articles, and their scientific background.

**Practices**	**Disciplines/scientific background**
Workshops/Moments to discuss and reflect on problem situations and knowledge to apply	Business Management [6, 7, 9, 16, 19, 25, 28], Sociology [3], Ergonomics [2, 12]
Digital communities/internal knowledge sharing platforms	Business Management [5, 8, 10, 11, 14, 18, 19, 21, 25, 26]
Training sessions	Business Management [6, 11, 16, 18, 20, 23, 24], Work Psychology [1], Ergonomics [17]
Mentoring	Business Management [13, 15, 19, 27, 28], Work Psychology [1]
Communities of practice	Business Management [14, 15, 22, 28]
Storytelling	Business Management [13, 15, 27]
Cognitive maps (maps of identification and relationship between work situations and knowledge)	Business Management [22]
Training program with video records (in the first and third person) of the activity to be transmitted	Ergonomics [4]
Repositories of documents and information	Business Management [13, 16, 23, 28]

In Table 3, it becomes evident that the most used practices are: implementation of moments to discuss and reflect on problem situations and knowledge to apply; development of digital communities/internal knowledge sharing platforms; and organization of training sessions (research question 2).

The results show that there are some practices shared by different disciplines, like moments to discuss and reflect on problems at work and knowledge to apply; training sessions; and mentoring.

### 3.3. Professionals responsible for the conduction of the programs and their role

Regarding those responsible for implementing the programs (research question 3), the majority were internal members of the companies, yet the departments are not identified. The remaining (13 studies) were implemented by external researchers, some with the support of internal departments. In some of the cases (five articles), pivotal figures were assumed as responsible for supporting or monitoring the implemented processes (e.g., direct leaders), which was pointed out as an added value for the pursuit of practices.

Most of the studies also involved key stakeholders, such as project managers and department managers, who took on the role of facilitators and leaders in the design and implementation of the initiatives. In only three of the studies was identified the presence of HR members (as participants), but it was only in two of these three studies that their support was requested to facilitate communication and increase the visibility of the initiatives implemented and to support the implementation of the program. This shows that HR do not appear to have a strong presence in the design or implementation of these processes.

### 3.4. What and how knowledge is managed or transmitted and duration of the programs

With regard to the type of knowledge that is transmitted and how (research question 4), it was noticed that the procedural, implicit or explicit knowledge are privileged in studies within the scope of Business Management; and the experiential and prudence knowledge are those privileged in studies within the scope of disciplines from Social Sciences.

It should be noted that, only in nine articles, references have been made to the type of knowledge to be transmitted/involved in actions of knowledge management or transmission: intention to make implicit knowledge explicit; knowledge considered critical for the company; experiential and prudence knowledge. Regarding the way the knowledge is transmitted, it was possible to identify the option for the extraction and storage of knowledge within the company and for the transmission and sharing of knowledge. In order to do so, the following data collection techniques were used to identify the knowledge: analysis of internal documentation (six articles), observations of work (three articles), semi-structured interviews, narrative and retrospective interviews, and collective interviews with actors with critical knowledge, focus groups for validation of content to be transmitted or identified and for analysis of implemented initiatives (12 articles); video records of the real activity and professional gestures (one article).

These results show that knowledge is not just procedural knowledge, but it is essentially secular/non-expert knowledge that links the questions of the effectiveness of professional gestures and of their safety, from the point of view of occupational health.

Regarding the duration of the mentioned implemented practices (research question 5), the majority of the studies indicate a 1-year period, approximately (six articles— year; one article—more than 2 years; one article— years) and one article indicates a duration of a few months; in the remaining articles, it was not possible to find this information.

Although the data on working conditions were not a primary concern in the research questions, the results revealed interesting data. The analysis of the presence/absence of the dimensions such as “working conditions” and “employee involvement” show that in most of the implemented programs, these dimensions do not appear to be considered or mentioned. In fact, and as another curious result, while “employee involvement” is mentioned explicitly in most of the articles, the “working conditions” only have visibility within the articles identified from the field of Work Psychology and Ergonomics.

### 3.5. Impact of the programs for workplaces at individual, group and organizational level

The findings reveal the implemented programs had an impact on the corresponding contexts (research question 6): (i) the role of leadership as a facilitator in the process of implementing the practices (five articles) and in developing a vision on knowledge management; (ii) the development of frameworks for knowledge sharing (and not so much in the transmission or development of those who will use that knowledge); (iii) the production of materials for the companies (e.g., procedure manual, digital platforms for knowledge sharing) (10 articles); (iv) and the identification of essencial learned lessons for the pursuit of practices.

The implemented practices had an impact at different levels in their contexts (in 16 of the 28 articles): at the individual level–for employees; at the group level–for teams; and at the organizational level–for the company and the business. At the individual level, for example, it was highlighted: (i) the feeling of recognition mentioned by the workers, for having been involved in the process, which boosted their learning and sharing of knowledge and confidence, in the way work is carried out; (ii) greater confidence during work and improvements in terms of autonomous problem-solving. At the group level, the impact that stood out was the promotion of collaboration between workers and the role assumed by the leadership. This proved to be a fundamental part in the management of practices and the role of conciliation, organization, conducting the process, and involving and valuing the different actors. At the organizational level, the following impacts were highlighted: the creation of a culture of knowledge sharing (through the created digital networks); the promotion of safety at work; and business expansion with the possibility to recruit and train new workers; as well as the impact on productivity/reduction of errors and times at work.

Despite these benefits and positive impact, some limitations were also identified: (i) content on digital platforms not being frequently updated, creating gaps between the information made available and that which is known; (ii) the perception that, sometimes, little priority is given to knowledge management; (iii) organizational conditions that affect the identification and sharing of knowledge (e.g., precarious employment; (iv) team instability; work intensification and increased workload); (v) workers not having enough time, in their daily work, to dedicate to these initiatives. On this last point, nine of the 28 studies mentioned time as an important dimension to be taken into account when implementing knowledge management practices (e.g., time to prepare, implement and update actions), in order to assure the quality and sustainability of the practices.

## 4. Discussion

### 4.1. Two complementary scientific approaches about knowledge-related practices

The analyzed studies reinforce the fact that the programs and practices related to the issues of workers' knowledge can be adopted by different scientific approaches, regardless of the global issues to address by companies. Yet, this systematic review supports the understanding that the way they are implemented in professional contexts assumes relatively distinct assumptions and analyses, where both knowledge management and knowledge transmission have their advantages, considering the respective scientific areas that underpin them.

The studies from Business Management (considering the knowledge management approach in its broadest sense, in which the perspectives of knowledge retention, transfer and sharing can be integrated) focus on a macro analysis of processes, through the extraction and storage of procedural, explicit or implicit knowledge. The way the knowledge impacts the productive processes is noticeable. This approach seems to demonstrate a positive impact on the production process, sharing of knowledge and business progress.

The studies from Work Psychology/Ergonomics (considering the knowledge transmission approach) focus on a micro analysis of the conditions in which the work is carried out and the transmission can occur. At this level, explicit priority is given to the necessary conditions to promote the actions of transmission and the identification of those that represent experiential knowledge. In this case, there seems to be a positive impact on the way workers are involved in processes and on how knowledge is transmitted and/or preserved in the contexts.

It is also noted that several techniques were used in a complementary manner (e.g., narrative interviews, construction of cognitive maps). However, the type of knowledge to be identified or transmitted was not emphasized or mentioned in the vast majority of the studies. It has also become clear that most practices are aimed at “white collar” workers and their use cannot be generalized to all functional areas (e.g., operational areas), due to the different characteristics and working conditions associated with different types of activity.

Despite the difficulty that workers have in verbalizing the acquired knowledge, namely experience-based knowledge (Oddone, 2007), the used techniques seem to help in structuring and raising awareness about knowledge and how to carry out the work, which becomes useful for the process of transmission. However, only in the studies from the additional records were working conditions considered (e.g., workload, pace of work, team instability). This corroborates results of previous studies which indicate that programs for the transmission and retention of knowledge are sometimes implemented without understanding the working conditions or the workers' perspectives (Joulain and Martin, 2013). These are elements that should be taken into account, in particular to guide the analysis and identification of knowledge, because specifically the workers' critical or implicit knowledge derives from practices that are accumulated over time. Knowledge is built on the intensity of the experiences in the context of work and on the actual characteristics and conditions of their activity (Lamari, 2010). Moreover, this discussion also reinforces the idea that the identification and transmission of knowledge, in order to be effective, must consider the conditions under which the work is done, since it is under these conditions that the transmission will take place.

We found that the design and implementation of the practices was undertaken mainly by internal members of the companies (e.g., managers). Although HR assume a fundamental role in professional contexts, with practices to promote the development and commitment of the organization and workers according to their needs (Staniewski, 2008), these actors were not integrated in the studies we found, which is a curious result. It is not clear why they are absent from these programs. Two interpretations are more likely. It may reveal either an absence of a collaborative and participative strategy for management or transmission of knowledge in organizations or a possible underappreciation of the role HR may assume in these contexts.

Finally, the impact of the programs (e.g., feeling of recognition, confidence, creation of a culture of knowledge sharing) helps to understand that the development of processes to deal with the worker's knowledge must be assumed as a strategic axis in work contexts. Furthermore, it should be seen not as a project with limited duration in time, but as a dynamic and evolutionary process (e.g., Haider, 2009), which also involves the allocation of time and resources.

With the additional records analyzed within the disciplines under the scope of Theory of Activity Research and social sciences (e.g., Work Psychology, Ergonomics), real work activity should be valued and transmission should take place as part of work activity. It can be achieved through action situations that are interpreted by workers, taking into account their life and work experience (e.g., Cloutier et al., 2012), maintaining a variable and evolutionary nature reconfigured according to the circumstances (Thébault, 2018). This last aspect is essential, since we have found that some practices implemented in the studies (e.g., static digital platforms; documents) do not contemplate the possibility of revisiting and updating the available contents, thus putting them at risk of becoming obsolete and of little use for learning and work performance.

While in the studies from the records identified through database searching the focus is mainly on a macro (organizational) level, seeking impact for the company and for productivity; in these disciplines the focus is mainly on a micro (individual and their collective) level. The analysis ends up being more focused on the workers, on the role they assume, on the valorisation of their experience and active participation in the process (e.g., Cloutier et al., 2012).

With regard to the methodologies used to prepare the initiatives and identify knowledge (e.g., implicit/critical), there are several techniques that are common among the different approaches exposed in this article, such as the use of communities of practice, holding training sessions, interviews, to name but a few. However, there are other elements that are privileged in these particular approaches: analysis of the real work activity, training sessions where the holders of knowledge are the protagonists; individual and collective interviews and self-confrontation interviews; video records of the real activity and professional gestures; and reflective workshops with workers. These approaches favor the recognition of workers and the identification of knowledge to be transmitted/managed. It should be noted that, due to the type of applied practices, they are typically carried out by members external to the companies. Still, there is the aim to train the companies' interlocutors, in order to make the use of these practices autonomous.

The explanation of the two scientific approaches complements the reflection on this subject with other perspectives and other stands on how to think and act concerning the relation between knowledge management (as a broad area within business management) and knowledge transmission (as a process most frequently studied in the field of work psychology and ergonomics) in workplaces. It is clear that there are common aspects on the theoretical backgrounds of these programs and there are also specific and structurally distinct ones. Although this is an unusual and quite complex dialogue, the incorporation of some articles of this nature has shown that there is potential to be gained from this articulation.

### 4.2. A conceptual framework to support management or transmission of knowledge in companies

The evidence and discussion from the systematic review supported that there are similarities and differences in the way workers' knowledge is treated and in the type of dimensions that are considered. Given the scientific problem of the absence of literature that illustrates the relationship between knowledge management (as a broad area in the scope of business management) and knowledge transmission (as a process often studied in work psychology and ergonomics) in the definition or implementation of activities or practices of management or transmission of knowledge, a theoretical framework is presented in this section. The framework is proposed to support the design and implementation of a management or transmission of knowledge program. It considers the branches of knowledge management and the knowledge transmission implicitly.

**Figure 2:**
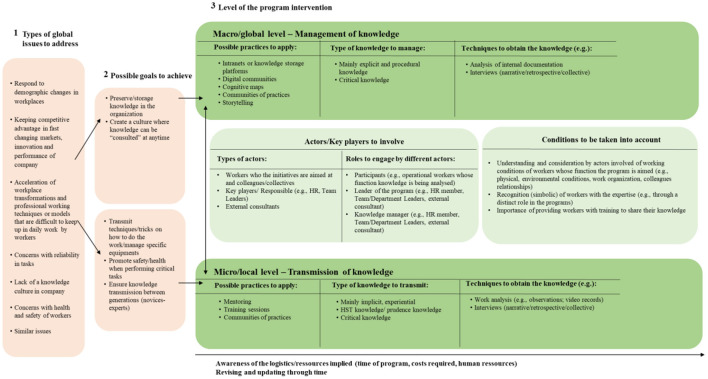
Theoretical framework for the design and implementation of a management or transmission of knowledge program.

This framework—shown in Figure 2—considers three main dimensions around the design and implementation of programs at two complementary levels. The first dimension corresponds to the possible global issues present in the workplaces. This dimension, which assumes a background role in the whole process, is important so that the company is clear about the reason behind the need to invest in a management or transmission of knowledge program. Once the issue is made explicit, the goals that the company wants to achieve should be addressed—second dimension -, through the clarification of the relationship between these two dimensions and the third one. The choice of the goals to achieve is not dependent on the global issue that the company wants to address. However, after the positioning in relation to the goals, the actions may be mainly in one of the two levels. In other words, there is a direct and consequent relationship between the second and third dimension: the level of action is dependent on the objective to be reached. On the contrary, there is no consequent relationship between the first and second dimension. The identification of the global issue does not necessarily induce the positioning in one of the two groups of possible goals to achieve.

In the third dimension, programs for management or transmission of knowledge may be at one of two levels (or at both, in a complementary form), according to the objectives the company wants to achieve: a macro/global program intervention can be developed, through the management of knowledge, or a micro/local program intervention, through the transmission of knowledge^2^. This is the dimension that determines the most appropriate level of action. Once this is defined, it becomes possible to think about the different elements encompassed in each of the two levels: the possible practices to apply, the possible techniques to obtain the knowledge and the type of knowledge to manage or transmit. These three elements are those that typically differentiate a knowledge management from a knowledge transmission program, as they take on different characteristics, yet allow the intended goals to be met.

Following this process, a fourth and fifth element that match the two levels of program intervention were considered: the key players to involve and the roles they assume, and the working conditions to consider during the design and implementation of programs. Regarding the key players, it is important to note that although there is a possibility of involving the same type of participants at both levels, they can take a more or less active role depending on the level of the intervention. The same applies to the conditions to be taken into account, that assume different levels of depth depending on the programs' purpose.

The framework also considers a temporal dimension, related to logistic aspects of the process. This dimension accompanies the entire process and corresponds to the awareness of the logistics/resources that should be implied in the implementation of such initiatives. It also underlines the importance of revising and updating the implemented initiatives as necessary, for instance, through the systematization of lessons learned from the program, to ensure their adequacy to the constant changes and work demands that arise in the work contexts. Consequently, it is then possible to introduce improvements that allow for the maintenance of practices over time.

It is worth noting that even if it is necessary to choose a focus (macro or micro) and that a transmission process does not automatically imply the management of knowledge in the company or vice versa, the approaches can be complementary. It is also possible to start a transmission process and later move to a management process (e.g., recovering the work done and organizing and storing it in the workplace); and it is also possible to start a management of knowledge process and move to a transmission one (e.g., by deepening the knowledge associated with a particular function or work technique that is embedded in the overall knowledge management process). This means that even though these two levels have different scientific backgrounds and purposes, both perspectives can benefit each other, thus contributing to the company's progress in terms of knowledge creation, development and achievement of global objectives. Furthermore, the framework is assumed to be dynamic, and not linear: it is not expected that after the identification of the global issues to address and the objectives to achieve, the steps to pursue will necessarily follow the order indicated here. In other words, the possible practices to apply, the possible techniques to obtain the knowledge and the type of knowledge to manage or transmit, are dimensions to be considered, but they can be defined in a dynamic and complementary way, and not necessarily one after the other.

In addition to the theoretical contribution of the framework, it also has a particular relevance for organizational practitioners, in particular to HR developers. It is believed that given the knowledge these professionals have on the organization/organizational systems and procedures and given the fundamental role they play in these contexts, they can foster the development and training of workers and knowledge (Metz et al., 2014; Jacobs, 2017). The results from the different articles show the structuring of the dimensions and the relationships among them also contribute to sustain the relevance of the proposed framework.

### 4.3. Conclusions, limitations, and practical implications

This article aimed to conceptualize a framework for the design and implementation of a knowledge management or transmission program. This was achieved through a systematic review that identified the management or transmission of knowledge programs carried out in professional contexts over the last 18 years.

Considering the absence of literature that illustrates the relationship between knowledge management as a broad area under the scope of the business management and knowledge transmission as a process under the scope of the work psychology and ergonomics perspective in the definition or implementation of activities or practices of management or transmission of knowledge, the conceptual framework provides inputs for reflection and possible action. It is oriented towards companies and, in particular, HR Departments, regarding the design and implementation of initiatives within this subject. Although this topic is not new for researchers or practitioners, no literature has been found on this point of view. The innovative character of this article lies in the fact that it systematizes the existing programs that have been put into practice and how it has been done. In addition, it conceptualizes the dimensions to consider (and relations between them) when designing and implementing a program of this nature, bringing different scientific perspectives into the discussion. However, the main limitation of the article is the number of databases in which the systematic review was conducted and the year option assumed for the articles search (2004), which may have excluded other articles that could complement the performed analysis. In addition, despite the consciously assumed theoretical positioning of privileging two scientific fields (business management; and work psychology/ergonomics) to address the scientific problem, it is understood that other complementary fields may not have been integrated.

From the analysis of the articles in the systematic review and the proposed framework, this study provides the following insights: (i) what defines the most appropriate practices or activities to implement is not the overall challenge, but rather the objective that the professional contexts want to achieve; (ii) knowledge management or transmission initiatives can have a positive impact at different levels (micro to macro), which is enhanced through the combination of several dimensions considered in the definition and/or implementation of the knowledge management or knowledge transmission programs (e.g. actors/key players; possible practices; type of knowledge; techniques to obtain the knowledge; time); (iii) HR still assumes a small to non-existent role in these knowledge management or transmission, despite being a department typically responsible for the training, learning and development of workers in companies.

Considering the results, the implementation of this type of initiatives can play an important role in the development of workers and in the professional contexts, as well as in meeting the companies' global challenges. Nevertheless, the systematic review also reveals that there are still aspects to be improved or studied on this matter, with regard to the design and implementation of these practices, in professional contexts or in scientific knowledge. For instance: (i) the apparent lack of involvement of HR Departments in these practices (or their reference/participation in studies); (ii) the fact there still seems to be scarcity of information or focus on the type of knowledge preserved or transmitted. It would be interesting to develop, in future studies, the issue of HR involvement and the role HR staff play, so as to deepen the analysis presented here and to further enhance the purposed framework. For example, to understand why these actors do not seem to have an active role in the design and implementation of the practices.

The study makes some advances for the scientific field and for practitioners. The first one is directed to the scientific community. The dialogue around complementary approaches on programs for management or transmission of knowledge has consolidated the reflection on this subject and reveals a strong support for the theoretical implications by crossing different perspectives and bringing scientific discourses closer together. As a consequence, both the discussion around this topic and the analysis are enhanced. A second one, directed at the practitioners, allows for advancements in their understanding of the concepts of knowledge management and knowledge transmission, therefore improving their professional practice by considering the real problems and needs, as well as the objectives set in the professional contexts. This also enlightens some practical implications, mainly by reinforcing the possibilities for those who intend to implement a program of this kind to act in professional contexts, based on scientific perspectives. This reveals the elasticity of the conducted work, that is, the quality of being adaptable to different contexts and local concerns, and the importance that theoretical works can have not only to the academic audience but also to practitioners and professional contexts.

## Data availability statement

The original contributions presented in the study are included in the article/Supplementary material, further inquiries can be directed to the corresponding author.

## Author contributions

CP conducted the systematic review research and wrote the first draft of the manuscript. CD and MS contributed to the design of the conceptual framework presented in the Discussion. All authors discussed the results and contributed to manuscript revision and final version, read, and approved the submitted version.

## Footnotes

^1^Knowledge incorporated into the professional experience and the activity itself, not perceptible to the organization, and that assumes an important role for the preservation of health and safety (Cru and Dejours, 1983).

^2^For further analysis please consider the following examples of studies that correspond mainly to a macro level intervention—e.g., 5, 10, 13, 14—and micro level intervention—e.g., 1, 2, 3, 4.
