# Supplementary material for: Conceptual framework for management or transmission of knowledge in companies: A systematic review
Source: Front Psychol. 2023 Apr 5;14:1124650. doi: 10.3389/fpsyg.2023.1124650 (PMC10116858; doi:10.3389/fpsyg.2023.1124650)
Supplement: Supplementary file 1 [file Table_1.docx]

Supplementary Material

Conceptual Framework for Management or Transmission of Knowledge in Companies: a systematic review

# Supplementary Table

Table S1. Identification of articles selected for the systematic review (organized by year).

|  | **Authors/Year** | **Aim of the study** | **Context and country** | **Journal /Scientific field** |
| --- | --- | --- | --- | --- |
| **[1]** | Santos et al. (2019) | Design and analyse a programme to welcome and train new employees privileging the transmission of know-how as a dynamic  process where learning is co-constructed in the course of the activity. | Chemical Industry, Portugal | Workplace Learning / Work Psychology |
| **[2]** | Thébault (2018) | Identification of main components of Knowledge transmission within a professional contexts. | Hospital, France | Formation et Emploi / Ergonomics |
| **[3]** | Dronne (2016) | Support the SMEs in the arts sector to better implement actions favourable to the transmission of professional knowledge and prudence between generations | Small and Medium Companies from Art sector, France | La Revue des conditions de travail / Sociology |
| **[4]** | Le Bellu (2016) | Develop a method to preserve experiential knowledge and promote sharing among newcomers and experts | Company of Electricity supplier, France | PISTES / Ergonomics |
| **[5]** | Shellum et al. (2016) | Design a knowledge management program to identify, store and disseminate clinical knowledge | Medical Clinic, United States of America | Learn Health Sys. / Business Management (BM) |
| **[6]** | Taib et al. (2016) | Develop a knowledge transfer programme to develop skills related to working with children with terminal illnesses | Foundation for people with disabilities and terminal illnesses, Malaysia | Bangladesh Journal of Medical Science / BM |
| **[7]** | Abdullah et al. (2015) | Transfer knowledge about the design and construction of structures using Freemasonry and increase the knowledge and use of this system in the construction industry, through the design of a knowledge transfer program | Consulting Firm, Malaysia | Jurnal Teknologi (Sciences & Engineering) / BM |
| **[8]** | Gamo-Sanchez and Cegarra-Navarro (2015) | Analyze the practices of knowledge management and processes in the area of engineering and maintenance of a small airport to provide effective services. | Small airport, Spain | Journal of Knowledge Management / BM |
| **[9]** | Pollack and Pollack (2015) | Demonstrate the use of the Kotters model in the implementation of a knowledge management program, aimed at knowledge retention considering the aging workforce | Financial and Insurance Company, Australia | Systemic Practice and Action Research / BM |
| **[10]** | Janes et al., (2014) | Explore a knowledge management program in a law firm that makes explicit the use of web 2.0 technologies to create a knowledge culture | Law firm, United Kingdom | Journal of Knowledge Management / BM |
| **[11]** | Parker and Hine (2014) | Understand how participation in "knowledge intermediaries" programs affects organizational learning capabilities | Small and Medium Enterprises in Agriculture and Manufacturing, Australia | European Planning Studies / BM |
| **[12]** | Cloutier et al. (2012) | Explore the conditions that support the transmission of professional and prudence knowledge, through the identification of characteristic Action Situations focused on the work context and the mobilization of knowledge in different activities as practice for KT | Film technicians, Homecare nurses, Food service helpers, Canada | *Études et recherches* (Rapport) / Ergonomics |
| **[13]** | Harvey (2012) | Understand how an organisation deals with the needs of generational transfer of knowledge and the strategy implemented for this purpose | Health and Social Services Centre, Canada | Journal of Knowledge Management / BM |
| **[14]** | Oluikpe (2012) | Explore the development of a knowledge management strategy in a bank | Bank, Nigeria | Journal of Knowledge Management / BM |
| **[15]** | Pollack (2012) | Review of a knowledge management programme whose approach emphasised visibility, support of seniors and enthusiasm for participation | Financial and Insurance Company, Australia | International Journal of Project Management / BM |
| **[16]** | Amalia and Nugroho (2011) | Explore the implementation of knowledge management in a multinational telecommunications company and analyze the factors that affect performance in the company | Multinational telecommunications company, Indonesia | Journal of Knowledge Management / BM |
| **[17]** | Antle et al. (2011) | Review of a knowledge transfer process of implementing a knife sharpening and steeling program into a poultry processing plant via a participatory ergonomics intervention | Poultry processing plant, Canada | Work / Ergonomics |
| **[18]** | Sandhawalia and Dalcher (2011) | Explore how knowledge management supports organizational practices in a company | Large software application company, United Kingdom | Journal of Knowledge Management / BM |
| **[19]** | Cegarra-Navarro and Cepeda-Carrión (2010) | Present a conceptual framework to guide learning in the context of home health care services | Home care unit - Regional hospital, Spain | Leadership in Health Services / BM |
| **[20]** | Seba and Rowley (2010) | Investigate strategies, policies, initiatives, programmes and practices relevant to the implementation of knowledge management in the UK police force. | Police forces, United Kingdom | Journal of Knowledge Management / BM |
| **[21]** | Haider (2009) | Explore success factors in implementing a knowledge management programme | SoftNetCo (software company), United Kingdom | Knowledge And Process Management / BM |
| **[22]** | Kwong and Lee (2009) | Explain the tacit knowledge of engineers in the field of technical reliability through narratives and cognitive mapping | Engineering Department of an airline, Hong Kong | Journal Of Knowledge Management / BM |
| **[23]** | Hofer-Alfeis (2008) | Exploring the "Leaving Expert Debriefing" knowledge management process in two professional contexts (SME and Large Enterprise) | SME and Large Enterprise (unidentified sector), Germany | Journal of Knowledge Management / BM |
| **[24]** | Makani (2008) | Identify the role that training for professional librarians plays in capturing, organizing and disseminating knowledge | International Federation of the Red Cross, Geneva | International Journal of Libraries and Information Studies / BM |
| **[25]** | Sánchez and Palacios (2008) | Understand how managers in manufacturing companies address the challenges of knowledge management | Repsol, Spain | Journal of Manufacturing Technology  Management / BM |
| **[26]** | Yam et al. (2007) | Explore how knowledge management can support an operational area of a networked enterprise in gaining competitiveness | Hasbro Far East Ltd, USA | Journal of Knowledge Management / BM |
| **[27]** | Holm et al. (2006) | Provide insights into the work of a consortium of companies related to space activities with respect to knowledge management architecture that is necessary to ensure a short and long term plan of knowledge management program | Space Industry Sector, USA | Journal of Knowledge Management / BM |
| **[28]** | Albert and Picq (2004) | Present a case study of an organisation that has successfully introduced knowledge management initiatives, promoting innovation and learning within the company | HP Lab Research Laboratory, USA | European Journal of Innovation Management / BM |
